# Integrating DNA Methylation and Gene Expression Data in the Development of the Soybean-*Bradyrhizobium* N_2_-Fixing Symbiosis

**DOI:** 10.3389/fmicb.2016.00518

**Published:** 2016-04-22

**Authors:** Austin G. Davis-Richardson, Jordan T. Russell, Raquel Dias, Andrew J. McKinlay, Ronald Canepa, Jennie R. Fagen, Kristin T. Rusoff, Jennifer C. Drew, Bryan Kolaczkowski, David W. Emerich, Eric W. Triplett

**Affiliations:** ^1^Microbiology and Cell Science Department, Institute of Food and Agricultural Sciences, University of FloridaGainesville, FL, USA; ^2^Biochemistry Department, University of MissouriColumbia, MO, USA

**Keywords:** epigenetics, DNA methyltransferase, root nodulation, nitrogen fixation, glycine max

## Abstract

Very little is known about the role of epigenetics in the differentiation of a bacterium from the free-living to the symbiotic state. Here genome-wide analysis of DNA methylation changes between these states is described using the model of symbiosis between soybean and its root nodule-forming, nitrogen-fixing symbiont, *Bradyrhizobium diazoefficiens*. PacBio resequencing of the *B. diazoefficiens* genome from both states revealed 43,061 sites recognized by five motifs with the potential to be methylated genome-wide. Of those sites, 3276 changed methylation states in 2921 genes or 35.5% of all genes in the genome. Over 10% of the methylation changes occurred within the symbiosis island that comprises 7.4% of the genome. The CCTTGAG motif was methylated only during symbiosis with 1361 adenosines methylated among the 1700 possible sites. Another 89 genes within the symbiotic island and 768 genes throughout the genome were found to have methylation and significant expression changes during symbiotic development. Of those, nine known symbiosis genes involved in all phases of symbiotic development including early infection events, nodule development, and nitrogenase production. These associations between methylation and expression changes in many *B. diazoefficiens* genes suggest an important role of the epigenome in bacterial differentiation to the symbiotic state.

## Introduction

Symbiotic associations between eukaryotes and bacteria are common and many model systems are well-studied. In many cases, the bacterium infects the eukaryote and becomes an intracellular resident of the host. In these cases, the bacterium differentiates to a quiescent form with a modified shape, evades host defenses, and modifies its gene expression to a phenotype that is beneficial to the host. In some models of symbioses, the processes of infection, and differentiation by the bacterium are well-studied. However, the degree to which epigenetics controls the infection and differentiation processes is unknown. Here, the role of epigenetics is examined within the symbiotic relationship between the nitrogen-fixing bacterium, *Bradyrhizobium diazoefficiens*, and its soybean host (*Glycine max* (L.) Merr.).

DNA adenine methylation in bacteria is well-studied and is known to play significant roles in the regulation of gene expression and in bacterial virulence (Heithoff et al., 1999; Low et al., 2001; Casadesus and Low, 2006). Gene expression by *B. diazoefficiens* changes dramatically during the transition between free-living growth and symbiosis (Franck et al., 2014). Given the large amount of gene expression changes that occur during symbiotic development, it is prudent to determine the extent of DNA methylation changes as well given the likely effect of DNA methylation on gene expression in this organism.

*B. diazoefficiens* (previously known as *B. japonicum*) is an alphaproteobacterium that infects soybean roots, induces the formation of root nodules, provides fixed nitrogen to the plant while colonizing root cortical cells, and differentiates into a non-dividing, symbiotic form. Much of the genetics, biochemistry, and physiology of this symbiosis is understood, but nothing is known about the possible epigenetic control of bacterial differentiation in this symbiosis.

Previous work showed methylation changes at the GANTC motif occur in the *Mesorhizobium loti* genome during formation of N_2_-fixing root nodules on *Lotus japonicus*, but their location in the genome was not identified and no correlation was made with gene expression changes (Ichida et al., 2007). Overexpression of the methyltransferase gene responsible for the deoxyadenosine methylation of the GANTC motif in *M. loti* showed delayed nodule development (Ichida et al., 2009).

Here, we assess differential methylation of DNA between the free-living and symbiotic states of five methylated motifs and their association with gene expression changes during symbiotic development at the whole genome level. Methylation and gene expression patterns across the whole genome are compared between free-living and symbiotic (bacteroid) *B. diazoefficiens* in order to determine the extent to which epigenetics controls the global transcriptional changes necessary to establish symbiosis.

## Materials and methods

### Bacterial and plant culture, bacteroid, and DNA isolation

*B. diazoefficiens* USDA 110 was cultured on Yeast Extract Mannitol (YEM) agar (Sherwood, 1972) at 28°C for 12 day. DNA was extracted from these cells using MoBio UltraClean DNA extraction kit (MoBio, USA). Soybean plants were cultured and inoculated with *B. diazoefficiens* USDA 110 and bacteroids isolated from root nodules as described previously (Franck et al., 2014). DNA was extracted and purified from bacteroids and free-living bacteria as described previously (Zhalnina et al., 2013).

### Whole genome shotgun sequencing of free-living *B. diazoefficiens*

Whole genome sequencing was done using the Pacific Biosciences (PacBio) platform with the P6-C4 chemistry (Pacific Biosciences, Menlo, CA, USA). A total of 3 and 12 Single molecule, Real-Time (SMRT) cells were sequenced for the free-living bacterium and endosymbiont, respectively, in order to obtain adequate quality scores for methylation analysis. As the DNA template used for PacBio sequencing is not amplified, all DNA epigenetic modifications remains intact. These modifications are detected by the PacBio RS II system by a change in the kinetics of replicating modified bases during the sequencing process (Flusberg et al., 2010).

Reads obtained from bacterial culture were assembled to generate a single contig using the HGAP_Assembly.3 protocol on the PacBio SMRT Analysis server, resulting in a single contig. The contig was circularized by finding the overlap between the 10-kb regions flanking the 5′- and 3′-ends of the genome using BLASTN (Altschul et al., 1990). The origin of replication was identified by locating the DnaA binding boxes (TGTTTCACG). Locating the origin of replication allowed circularization of the genome, hereafter referred to as the reference genome.

The *B. diazoefficiens* genome was assembled and the methylation events and motifs called as described previously (Clark et al., 2012; Leonard et al., 2014). Re-analysis of gene expression data from Franck et al. (2014) was done using the LIMMA R package (Smyth, 2005). Differential gene expression *p*-values were adjusted for multiple hypothesis testing (Benjamini and Hochberg, 1995) and significance was considered at adjusted *p* < 0.001. To combine DNA methylation and expression data, the annotation of the reference *B. japonicum* genome (Göttfert et al., 2001; Kaneko et al., 2002; Delamuta et al., 2013) from RhizoBase (Fujisawa et al., 2014) was used to match the “bl_number” identifiers present in the expression data. Methylation events by motif and gene were then combined with gene expression data to form a single table by gene (Dataset S1).

### Methylation events and motifs calling

To obtain DNA methylation data and identify methylation motifs, the PacBio Motif and Methylation analysis protocol was performed using all reads obtained from the free-living culture, and all whitelisted reads for the bacteroid endosymbiont. Motifs were determined by the motif and methylation protocol (Altschul et al., 1990; Leonard et al., 2014). Methylation events that did not pass the minimum requirements recommended by PacBio (coverage ≥ 25 and quality score ≥ 30) were excluded from further analysis. In PacBIo sequencing, the kinetics of base incorporation changes with base modification. These kinetic differences identify the particular base modified and the location of the modification on the base.

### Differential gene expression analysis

Raw RNA microarray data from Franck et al. (2014) was retrieved from the Gene Expression Omnibus (Edgar et al., 2002) project GSE60279 in GenePix® (Molecular Devices, USA) format. Expression data was processed and analyzed using the LIMMA package in R (Smyth, 2005) to normalize for within and between microarray differences using the quantile and reduce methods, respectively; and to calculate the significance and magnitude (log_2_ of fold change) of differential gene expression between free-living and endosymbiont *B. diazoefficiens*. Differential gene expression *p*-values were adjusted for multiple hypothesis testing (Benjamini and Hochberg, 1995) and significance was considered at adjusted *p* < 0.001. Relative expression data from multiple microarray spots were averaged by taking the mean.

To obtain functional enzyme classifications, translated sequences were identified in the md5NR database (Wilke et al., 2012) using BLASTP and taking the best hit with high identity (*e* < 10^−5^, >90% identity). The sequences were then associated with hierarchical KEGG Orthologies (KO) in the KEGG database (Kanehisa and Goto, 2000). Data integration was performed using Python, IPython notebooks (Pérez and Granger, 2007), and Pandas (http://pandas.pydata.org/). Source code used in the analyses is available from https://github.com/triplett/bradyrhizobium-methylome-2015.

### Integration of gene expression and methylation analysis

Methylation events were grouped by gene into their respective coding or upstream non-coding regions as determined by the NCBI annotation. The number of each motif in the coding and 5′ regions of each gene were identified using regular expressions. Methylation events by motif and gene were then combined with gene expression data to form a single table with gene expression and methylation by gene (Table S1).

To obtain functional classifications of enzymes, translated amino acid sequences were identified in the md5NR database (Wilke et al., 2012) using BLASTP and taking the best hit with high identity (*e* < 10^−5^, >90% identity). The md5 checksums from the md5NR database were used to associate amino acid sequences with hierarchical KEGG Orthologies (KO) in the KEGG database (Kanehisa and Goto, 2000).

For each of the five motifs, DNA Methylation events were tallied between free and endosymbiotic states within the entire genome, the endosymbiotic region, and the remaining genome. Methylation events were compared within regions between coding regions and non-coding (5′-UTR) regions by their corresponding gene. Genes with differential methylation in their coding or upstream region which also had changes in gene expression were filtered to produce a list of genes potentially regulated by DNA methylation.

### Availability of source code and data

The resequenced *B. diazoefficiens* USDA 110 genome is available under accession Genbank no. CP011360. All raw reads are available from the NCBI Short Read Archive (SRA SUB1015301). More detail on methods is found in Supplemental Information.

Analysis of DNA methylation data and integration with expression data was performed using Python, IPython notebooks (Edgar et al., 2002), and Pandas (http://pandas.pydata.org/). Source code used in the analyses is freely available under the MIT open source license from https://github.com/triplett/bradyrhizobium-methylome-2015. Combined methylation, expression, and functional annotation for each of the methylated motifs are found in Table S1.

## Results

To study the DNA methylation pattern in *B. diazoefficiens*, DNA samples from a free-living culture and bacteroids were sequenced using the PacBio platform. For the free-living culture, 2.93-Gbp was obtained in 211,890 reads post quality filtering. Reads had a mean read length of 13,848. Reads were assembled to yield a single 9,113,303-bp contig with an average coverage of 258.85x. The resulting contig was circularized and set to start at the origin of replication resulting in a finished contig length of 9,106,064-bp, 236 bp longer than the previous closed reference of length 9,105,828-bp (NC_004463.1, Göttfert et al., 2001; Kaneko et al., 2002). The finished genome was aligned to the previously published genome using MAUVE (Darling et al., 2004) and found to contain only 48 SNPs. As both genomes were obtained from the same strain, any differences in genome sequence are likely to be sequencing errors and not real polymorphisms.

Of the 698,750 post-filter reads with an average read length of 9474 bases from the 12 PacBio SMRT cells sequenced using DNA extracted from soybean nodules inoculated with *B. diazoefficiens*, 225,991 (32.3%) mapped to the finished reference with an average concordance of 99.23% and average fold coverage of 331.12. Very little contamination from soybean DNA was observed: only 2725 bp (0.39%) by mapping to the *Glycine max* reference genome (NCBI RefSeq GCF_000004515.3) using the PacBio RS_Resequencing.1 protocol.

The location of the 681-kb symbiosis island was determined to be between genomic coordinates 1,000,419-bp and 1,681,419 on the new genome reference. These coordinates were obtained by BLASTing 1-kb sequences from the regions flanking the symbiosis island on the original reference against the new reference. A drop in GC-content within this region confirmed its location as previously described (3, 4).

### NCBI/KEGG annotation

Using the NCBI annotation server, 8265 protein-coding genes were identified. Of these, 8214 had significant matches in the MD5NR database using BLASTP (Altschul et al., 1990) with an *e* < 10^−5^, 3278 proteins (39.7% of total) had a corresponding KEGG Orthology annotation.

### Extent of methylation observed from each motif

Nearly all of the CAY(N)_6_CTC and GAGA(N)_6_RTG motifs were methylated in culture, but the frequency of methylation in these two motifs declined to 86.1 and 82.6% in the CAY(N)_6_CTC and GAGA(N)_6_RTG motifs, respectively, in the bacteroids (Table 1). This difference represents a total of 651 fewer methylated sites during symbiosis from these motifs alone.

**Table 1:** **Motif summary table for the PacBio sequencing SMRT cells of the (A) free-living *B. diazoefficiens* cells and (B) bacteroids from root nodules**.

**Motifs**	**Modified position**	**Type**	**Motifs methylated**		**Number of motifs**	**Mean QV**	**Mean coverage**	**Mean IPD ratio**	**Objective score**
			**%**	**#**					
**(A) FREE-LIVING BACTERIA**									
GANTC	2	m6A	99.77	33,118	33,196	136.16	129.93	4.61	4,499,647
CRAGGAT	6	m6A	99.95	4003	4005	196.26	130.19	6.29	720,736
CRAGGAT	1	m4C	15.31	613	4005	68.45	126.62	2.42	7749
GAGA(N)_6_RTG	4	m6A	100.0	2080	2080	182.73	129.81	5.73	359,565
CAY(N)_6_TCTC	2	m6A	99.95	2079	2080	190.17	130.22	6.13	395,193
**(B) BACTEROIDS**									
GANTC	2	m6A	97.46	32,353	33,196	84.99	62.89	5.07	26,86,751
CCTTGAG	6	m6A	80.06	1361	1700	57.77	66.75	4.07	61,541
CRAGGAT	6	m6A	87.49	3504	4005	53.47	64.67	3.46	144,719
GAGA(N)_6_RTG	4	m6A	82.60	1718	2080	50.72	63.95	3.27	67,049
CAY(N)_6_TCTC	2	m6A	86.06	1790	2080	50.69	64.13	3.37	79,264

*Where a motif is present in once condition and not the other, that motif was methylated only under one condition*.

In culture, 99.76% of GANTC motifs are methylated compared to 97.46% GANTC motifs during symbiosis (Table 1). This difference represents 765 fewer methylated sites during symbiosis compared to free-living culture.

All but two of the 4005 CRAGGAT motifs were methylated at the 6th adenosine position when the bacterium was cultured while, in the nodule, only 87.5% of these 4005 CRAGGAT motifs were methylated (Table 1). This represents 499 fewer methylated sites during symbiosis compared to culture.

The CCTTGAG motif was only methylated during symbiosis, where the m6A is methylated at the 6th position at over 80%, or 1361, of the sites (Table 1). This motif is methylated in 5′ upstream untranslated or coding regions of 1410 genes, 116 of which are in the symbiosis island. Of those 1410 genes, 398 genes (of which 36 are in the symbiosis island) are also significantly changed in gene expression.

### Genome wide differential methylation

A total of 3276 methylation changes were observed in five DNA motifs between the free-living and symbiotic states. PacBio SMRT Analysis identified that m6A methylation events at the GANTC, CRAGGAT, CAY(N)_6_CTC, and GAGA(N)_6_RTG motifs at positions 2, 6, 2, and 4, respectively, in the free-living culture (Table 1). A small amount of m4C methylation was observed at the 1st position of the CRAGGAT motif (Table 1) but these methylations are not discussed here since the cytosine methylation did not pass the minimum PacBio quality score requirement (*Q* > 30).

**Figure 1:**
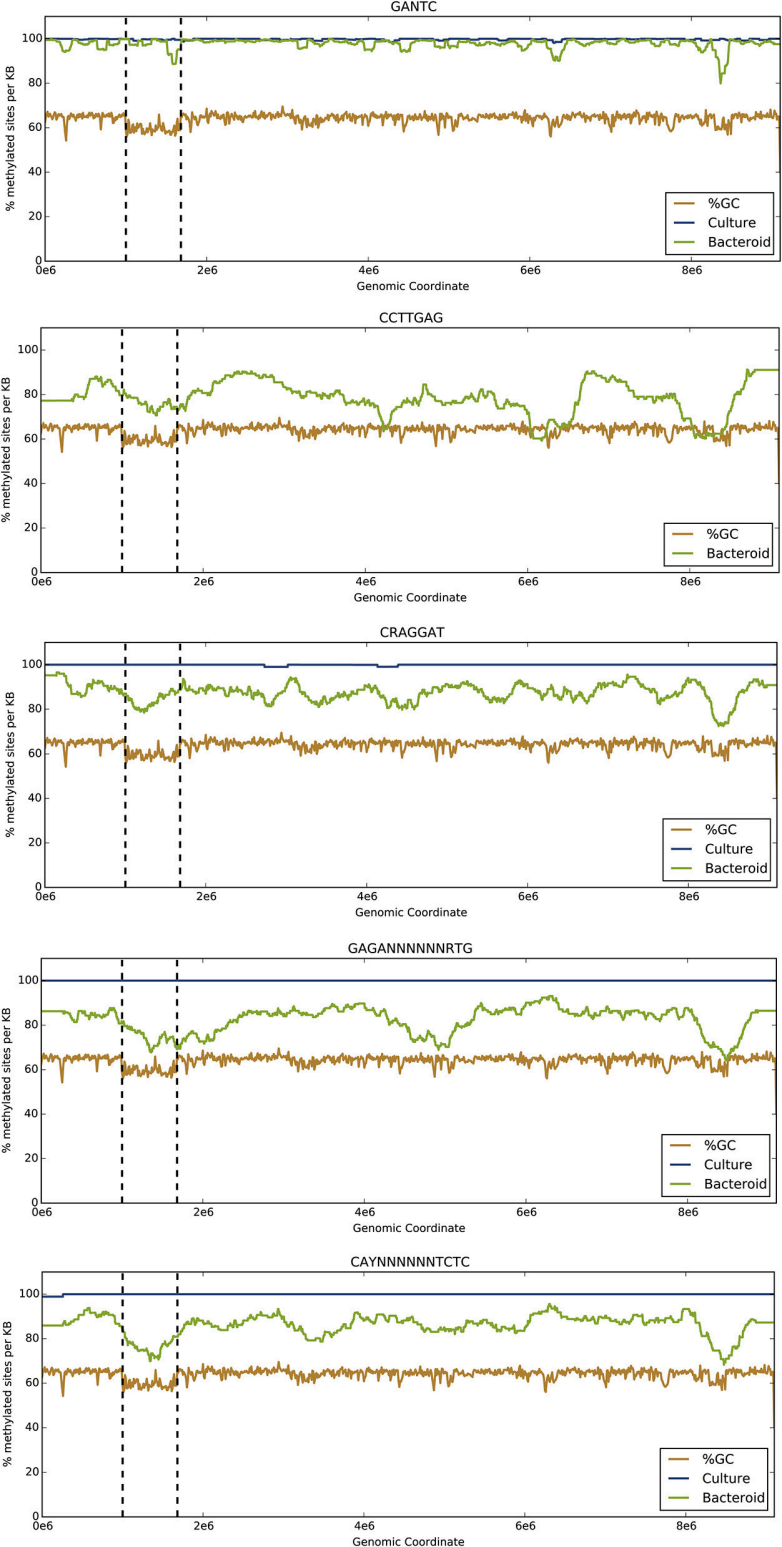
**The proportion of methylated sites changes between free-living and endosymbiotic states along the entire *B. diazoefficiens* genome**. The y-axis represents the percent of motif sites that are methylated in the free-living and bacteroid states. The brown line represents average percent GC content in the same sliding windows. The blue and green lines refer to % methylation across the genome in the free-living and bacteroid states, respectively. The vertical dotted lines delimit the symbiosis region.

The methylation motif summary tables are provided for the genome under free-living (Table 1A) and symbiotic conditions (Table 1B). To visualize differential DNA methylation between free-living and endosymbiont bacteroid states for each motif, the number of methylated motifs per total motifs was averaged over overlapping 1-kb sliding windows (Figure 1). Average GC content was averaged across the genome in the same manner to highlight the symbiosis region.

All motifs showed increased methylation across the genome in the free-living culture compared to the endosymbiont except for the CCTTGAG motif (Table 1, Figure 1). Global methylation for GANTC was similar with only a 2.3% decrease in the endosymbiont (Table S1, Figure 1). Methylation of the CRAGGAT, GAGA(N)_6_RTG, and CAY(N)_6_TCTC motifs decreased by 12.5, 17.4, and 13.9%, respectively, during the transition from the free-living to the symbiotic state (Table S1). Methylation of the CCTTGAG motif was only observed in symbiosis.

Of the five motifs identified, GANTC was by far the most common with 33,196 sites across the genome, including 3159 (9.51%) in the symbiosis island (Table S1). Methylation of the GANTC motif was similar between coding regions and upstream, untranscribed regions (5′-UTRs) per kb of DNA.

The CRAGGAT motif (4005 sites, Table 1, Figure 1) showed 12.5% reduced methylation in the symbiont (Table 1). A 6.9% decrease in 5′-UTR methylation per kb was observed within the symbiosis region compared to the rest of the genome. Methylation of the GAGA(N)_6_RTG motif was reduced by 17.4% across the genome for the symbiont (Table 1, Figure 1). A more modest decline in methylation of 13.9% was observed during symbiosis with the CAY(N)_6_TCTC motif (Tables 1, 2, Figure 1). Decreased methylation by between 4 and 7% in the 5′-UTR regions compared to coding regions was observed in all parts of the genome per kb (Table S1).

**Table 2:** **Summary of the seven genes proposed in the *Bradyrhizobium diazoefficiens* USDA 110 genome likely involved in the motif methylation described here**.

**Predicted motif**	**Rhizobase protein, gene**	**NCBI reference**	**Annotation**
CRAGGAT	blr0865	WP_011083687.1	Type IIG methyltransferase
GANTC	bll2509, ccrM	WP_011085296.1	CcrM modification methylase
CAY(N)_6_CTC/GAGA(N)_6_RTG	bll5012, hsdR	WP_011087773.1	Type 1 restriction modification system subunit R
CAY(N)_6_CTC/GAGA(N)_6_RTG	bll5013, hsdS	WP_011087774.1	Type 1 restriction modification system subunit S
CAY(N)_6_CTC/GAGA(N)_6_RTG	bll5014, hsdM	WP_011087775.1	Type 1 restriction modification system subunit M
CCTTGAG	bll0221	WP_011083053.1	Type II methyltransferase

The CCTTGAG motif was unique in that it is the only motif where methylation occurred exclusively in the symbiotic state. Of the 1700 sites of this motif in the genome, 1361 (80%) are methylated in the symbiont. Within the bacteroid, CCTTGAG showed a 12% decrease across the genome in 5′-UTR region methylation per kb compared to coding regions (Table S1). An increase in 5′-UTR methylation was observed within the symbiosis region (5%) compared to 1.6% in the remaining genome, opposite of the trend observed in CRAGGAT methylation (Table S1).

### Gene expression

**Figure 2:**
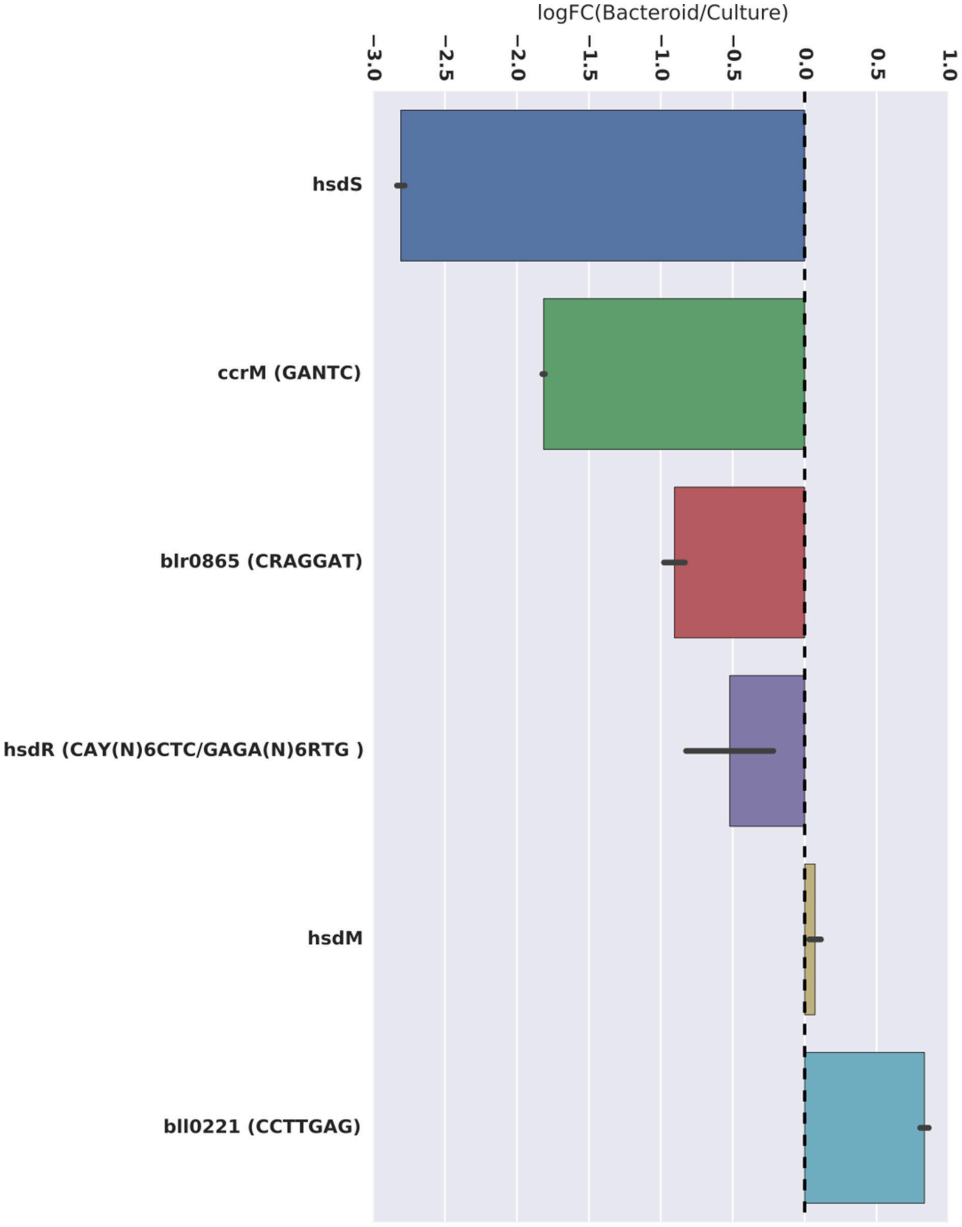
**Differential gene expression of *Bradyrhizobium diazoefficiens* during endosymbiosis**. The y-axis represents normalized relative expression values on a logarithmic scale, and the x-axis represents the position along the genome. Normalized expression values greater than 1 are more highly expressed in the free-living culture state and values < 1 are more expressed in during symbiosis. The shaded gray line represents expression across the genome and red dots represent genes that showed significant differential expression. Dotted vertical lines delimit the symbiosis region.

As expected, re-analysis of expression data yielded very similar results to those of Franck et al. (2014). A genome-wide representation of the change in gene expression between free-living and bacteroid states shows the increased expression of many genes within the symbiotic island during symbiosis (Figure 2). Overall, a greater number of genes had significantly increased expression during culture (1382, 16.81% of total) than in the bacteroid (800, 9.73% of total). The trend was the opposite for the expression of genes within the symbiosis region, with the bacteroid having more genes with increased expression (200, 34.31%) compared to the free-living culture (27, 7.09%). Of the genes that had increased expression in the bacteroid, 14 were involved in symbiosis according to the Rhizobase designation including *nolA, nolY, nolZ, LysR* family transcriptional regulator, *nodY*, and *noeE*.

### Associations between DNA methylation and gene expression

Over the entire genome, 768 genes changed in methylation states and in gene expression during differentiation to symbiosis (Table S2). This result represents 35.5% of all genes that show statistically significant changes in gene expression and 9.3% of all genes in the genome.

Within the symbiosis island, 15.3% of the 583 genes are both differentially expressed and methylated (Table S2). Of those 89 genes, only 7 have a known symbiotic role and all of those show increased gene expression during symbiosis. Of those 89 genes, 78 genes show increased gene expression while the remaining 11 are more expressed in the free-living bacterium. Of the 89 genes that are differentially methylated and expressed under symbiotic conditions, 82 genes have no known symbiotic function and 47 genes are classified as hypothetical (Table S2). Of the 11 genes with decreased expression in the symbiotic island, seven are annotated as hypothetical genes.

### Genes differentially methylated and expressed outside of the symbiosis island

In the genome, outside of the symbiosis region, 220 and 438 genes were more highly expressed in the endosymbiont and free-living bacteria, respectively. These genes can be classified into a wide range of KEGG categories including metabolism, environmental signal processing, genetic information processing, and cellular processes (Dataset S1).

## Discussion

### Integration of methylation and gene expression

Integration of methylation data with gene expression data from the same samples was done to get a first glimpse at the role of epigenetics in the symbiotic development of a bacterial strain. The 89 genes in the symbiotic island that showed differential expression and methylation patterns between the free-living and symbiotic states were of particular interest. As DNA methylation often affects gene expression, those 89 genes may be at least partially under epigenetic control and worthy of further investigation. Of those 89 genes, nearly 88% showed increased expression in the nodule while the remaining genes showed decreased expression in the nodule. Half of all 89 genes are classified as hypothetical, which suggests that future functional characterization of these genes may provide novel insight into the role of epigenetics in symbiotic development.

### Methylation, gene expression, and symbiosis

The remarkable aspect of the methylation changes observed within known symbiosis genes is that methylation and gene expression changes are observed simultaneously in genes involved in many phases of nodule development including sulfurylation of the Nod factor (*noeE*), efficiency of nodule development (*nfeC*), number of infection events (*nolY*), maturation of the nitrogenase complex (*nifB, nifE, nifX*, and *nifZ*), and the development of symbiotic nitrogen fixation (*fixA* and *fixC*).

These results suggest that symbiotic differentiation of the bacterial cell may be under partial epigenetic control. This conclusion is based on the strong differences in gene expression associated with methylation changes both within and outside the symbiosis region of the genome. In order to test this hypothesis, methylation, and expression need to be measured using more replicates across a time-series as the free-living culture transforms into the bacteroid state in order to capture enough data to sufficiently correlate methylation events with specific gene regulation events. In addition, knock-outs of the methyltransferases are needed to assess potential impacts on the symbiotic phenotype. As the GANTC methyltransferase, CcrM, is an essential gene (Kahng and Shapiro, 2001; Gonzalez and Collier, 2013), mutants with increased expression, or modification of specific motif sites thought to be involved in gene regulation, are needed. However, given what is known in other alphaproteobacteria, the GANTC motif is probably methylated by CcrM in *B. diazoefficiens* as well.

The CAY(N)_6_CTC/GAGA(N)_6_RTG motif is typical in structure for modification by a type 1 restriction modification system such as the *hsdRSM* system in *B. diazoefficiens*. Although CAY(N)_6_CTC/GAGA(N)_6_RTG represents two strands of the same motif, PacBio sequencing shows that different bases are methylated on each strand (Table 1). The adenosines in positions 2 and 4 are methylated in C**A**Y(N)_6_CTC and in GAG**A**(N)_6_RTG (in bold), respectively. This may explain why different levels of methylation are observed on each strand under symbiotic conditions (Table 1). The kinetics of the methylation reaction may differ on the two strands.

### Specific symbiosis functions with altered gene expression and methylation patterns

The observation that 3276 methylation changes are observed to occur in this bacterial genome during symbiotic development is significant and suggests many worthy experiments for the future. These methylation changes often co-occur with gene expression changes, including 24 methylation changes observed in seven known symbiosis genes whose expression increases in the nodule. These seven genes are involved in most of the stages in the development of nitrogen-fixing root nodules. Similar epigenetic changes may occur in other symbioses or other host-microbe interactions.

Nearly all of the genes known to be necessary for infection (nod), nitrogen fixation (*nif*, *fix*), and hydrogen oxidation (*hup, hyp*, and *hox*) within the *B. diazoefficiens* genome are contained within a 681-kb symbiosis region (Göttfert et al., 2001; Kaneko et al., 2002). Hydrogen oxidation improves the efficiency of nitrogen fixation by oxidizing an obligate product of the nitrogenase reaction, H_2_, to recover the electrons for ATP production (Albrecht et al., 1979; Maier and Triplett, 1996). Despite all of the attention given to the symbiosis island over the years, more than half of the predicted coding regions in this island are still annotated as hypothetical, which is higher than the rest of the genome in which only a third of the genes are of unknown function. This high proportion of genes with unknown function suggests that there is much more to learn about symbiotic development in this organism.

Within the symbiosis island, there are 9 genes with known roles in symbiosis that were differentially expressed and methylated. All of these genes had increased expression during symbiosis (Table 3). Of this set, two are nitrogen fixation (*nif*) genes involved in the maturation of dinitrogenase, which is also referred to as the MoFe protein. Both of these genes (*nifB, nifE, nifX*, and *nifZ*) play essential roles in the synthesis of the FeMo cofactor of dinitrogenase (Shah et al., 1994; Huang et al., 1999; Hernandez et al., 2007).

**Table 3:** **Gene products of known symbiosis genes with statistically higher gene expression and methylation changes**.

**Protein**	**Motifs changed with symbiosis**	**No. methylation changes**	**logFC**	**Protein function**
NifE (blr1745)	GANTC	1	3.06	FeMoco synthesis (Hernandez et al., 2007)
NifX (blr1747)	CRAGGAT	1	5.49	FeMoco synthesis (Hernandez et al., 2007)
NifZ (blr1761)	GANTC	1	4.60	P cluster synthesis for FeMoco (Huang et al., 1999)
NifB (blr1759)	CRAGGAT, GANTC	4	5.06	FeMoco synthesis (Shah et al., 1994)
FixC (blr1774)	CAY(N)_6_TCTC, CRAGGAT	3	4.55	Required for N_2_ fixation (Gubler and Hennecke, 1986)
NolY (bll2016)	CAY(N)_6_TCTC, CRAGGAT	3	1.23	Fewer infection events (Dockendorff et al., 1994)
FixA (blr2038)	CCTTGAG	1	0.89	Flavoprotein required for N_2_ fixation (Gubler and Hennecke, 1988; Weidenhaupt et al., 1996)
NfeC (bll2067)	CCTTGAG	1	5.73	Mutant delayed in nodulation (Chun and Stacey, 1994)
NoeE (blr2073)	GANTC, CRAGGAT	13	0.78	Sulfotransferase involved in Nod factor decoration (Vasquez et al., 1993; Hanin et al., 1997; Quesada-Vincens et al., 1998)

The gene products FixA and FixC are essential for symbiotic nitrogen fixation (Gubler and Hennecke, 1986, 1988; Weidenhaupt et al., 1996). NolY is needed for an optimal number of infection events resulting in higher nodule number (Dockendorff et al., 1994). Of particular interest are genes with homology to NoeI, a SAM-dependent methyltransferase. NoeE is a sulfotransferase that modifies the Nod factor (Vasquez et al., 1993; Hanin et al., 1997; Quesada-Vincens et al., 1998). A mutation in NfeC causes a delay in nodulation.

Three of these genes, *fixC, nolY*, and *noeE*, are differentially methylated at two distinct motifs (Table 3). Four of these genes, *nifB, fixC, nolY*, and *noeE*, are differentially methylated at 2 or more sites with *noeE* seeing methylation changes at 13 sites.

For each gene throughout the genome, each motif was examined for the presence or absence of methylation. This was done in the untranscribed region upstream of each gene as well as the coding region. Of the 9 known symbiosis genes with changes in methylation during symbiosis, 7 genes had changes in coding regions and 4 had changes in the upstream untranscribed regions. Of the 28 total changes observed in these seven genes, 14 changes were in the coding regions, and 14 changes were in upstream untranscribed regions. Most of the changes in the untranscribed region (11 of 14) were upstream of *noeE* while *nifB* had five changes, which represents the most changes in the coding region of any of these symbiosis genes.

### Identification of putative methyltransferases

**Figure 3:**
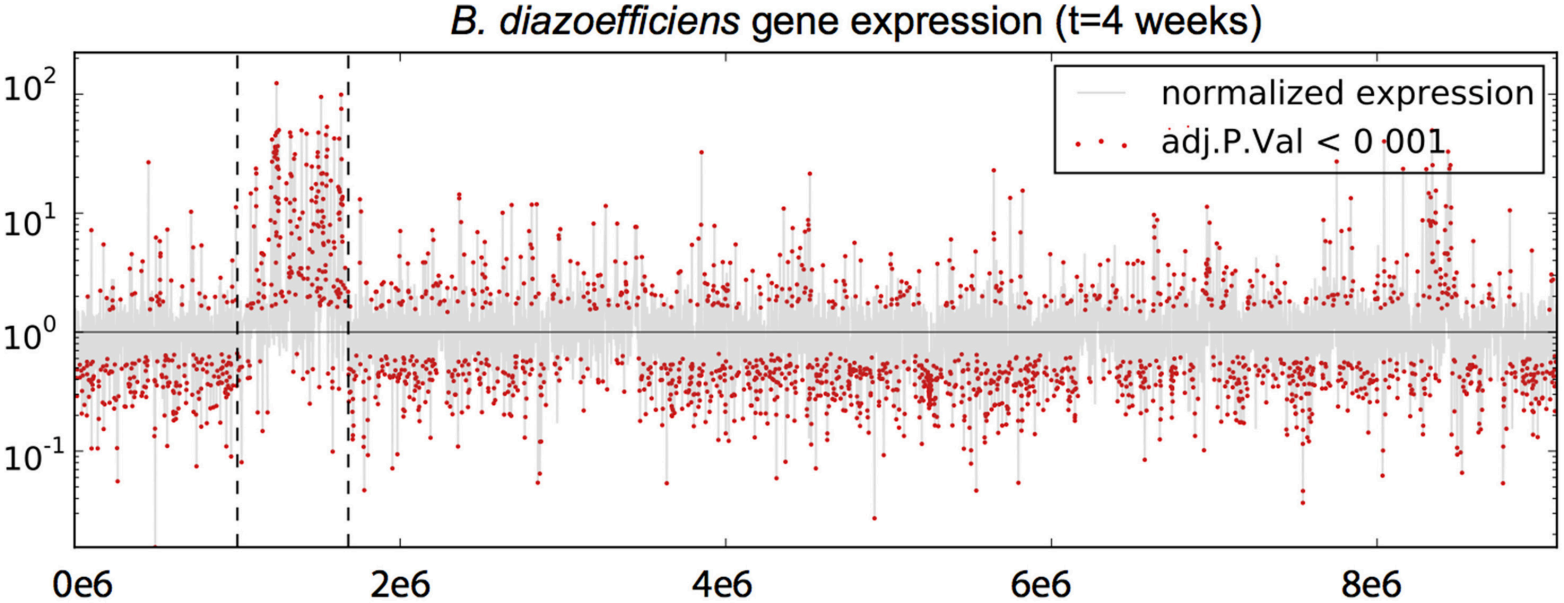
**Differential expression of the *ccrM* and the other potential DNA methyltransferases identified**. Average log relative fold-change expression is represented by the x-axis (LogFC > 1 = higher expression in symbiosis region). All potential methyltransferases with differential expression had decreased expression in the bacteroid. Significance of differential expression is denoted with asterisks (^***^ = adj. *p* < 0.001; ^**^ = adj. *p* < 0.01).

The CAY(N)_6_CTC and GAGA(N)_6_RTG motifs are palindromic and represent two different adenosine methylations, at the 2nd and 4th positions, respectively, of the same double-stranded motif. This motif structure is likely recognized by a type 1 restriction modification system (Loenen et al., 2013). The genes coding this system in *B. diazoefficiens* are *hsdRSM* (bll5012, bll5013, and bll5014, Table 2). Two of these genes, *hsdR* and *hsdS*, are significantly more expressed in free-living conditions compared to symbiotic conditions, which is consistent with the higher level of methylation of this motif in culture (Figure 3). The third gene, *hsdM*, is equally expressed in both culture and the nodule (Figure 3).

The GANTC motif is palindromic and is m6A methylated at position 2. The protein likely responsible for this methylation, CcrM (Table 2), is well studied in the alphaproteobacteria and is an essential gene necessary for cell division in *Caulobacter* and *Agrobacterium* (Kahng and Shapiro, 2001; Gonzalez and Collier, 2013). More GANTC methylation and *ccrM* gene expression are observed in culture than in the nodule.

A type IIG methyltransferase, coded by the blr0865 gene in the *B. diazoefficiens* genome and expressed significantly more in culture than in the nodule, is predicted to be the enzyme responsible for CRAGGAT methylation (Table 2, Figure 3). The rationale for this assignment is that blr0865 is the only N6 methyltransferase in the genome that is differentially expressed in culture. However, this hypothesis needs testing by mutagenesis.

### A symbiosis-specific methylated motif and putative methyltransferase responsible

Perhaps the most striking result of this work is the discovery of a symbiosis-specific methylated motif. A total of 1361 CCTTGAG motif sites are m6A methylated only during symbiosis. A type 2 methyltransferase coded by bll0221 may be enzyme responsible, as it is the only N6 methyltransferase that is significantly more expressed in the nodule (Table 2, Figure 3). Mutagenesis of bll0221 is required to test this notion. The impact of the CCTTGAG motif on this symbiosis could be important for identifying novel genes involved in symbiosis that were previously not known to play a role.

## Summary

Methylation analysis of whole bacterial genomes prior to and during symbiosis coupled with gene expression analysis in the same samples can lead to the discovery of hypothetical genes that may be involved in symbiosis. Symbiosis-specific motifs may be present in other symbiotic systems and their role in bacterial development toward symbiosis is worthy of future investigation.

## Author contributions

AD led the data analysis and contributed to the writing. JR, RD, and AM helped with the data analysis. JF and KR did the DNA extraction and helped with the design of the project. JD and BK contributed to the concepts and the writing. DE co-led the project with ET. ET did a large portion of the writing.

### Conflict of interest statement

The authors declare that the research was conducted in the absence of any commercial or financial relationships that could be construed as a potential conflict of interest.
