# Corrigendum: Integrating DNA Methylation and Gene Expression Data in the Development of the Soybean-*Bradyrhizobium* N_2_-Fixing Symbiosis

**DOI:** 10.3389/fmicb.2016.00952

**Published:** 2016-06-15

**Authors:** Austin G. Davis-Richardson, Jordan T. Russell, Raquel Dias, Andrew J. McKinlay, Ronald Canepa, Jennie R. Fagen, Kristin T. Rusoff, Jennifer C. Drew, Bryan Kolaczkowski, David W. Emerich, Eric W. Triplett

**Affiliations:** ^1^Microbiology and Cell Science Department, Institute of Food and Agricultural Sciences, University of FloridaGainesville, FL, USA; ^2^Biochemistry Department, University of MissouriColumbia, MO, USA

**Keywords:** epigenetics, DNA methyltransferase, root nodulation, nitrogen fixation, glycine max

Reason for Corrigendum:

In the paragraph titled “**Availability of Source Code and Data**”:

The published SRA accession number “(SRA SUB1015301)” is only a temporary submission ID and is not the permanent accession. This should be replaced with the correct SRA accession number “(SRA SRP061658).”

Please accept the sincerest apologies from the authors regarding this error. This correction does not impact the scientific conclusions of the article in any way.

## Author contributions

JR is responsible for composing and submitting this correction.

### Conflict of interest statement

The authors declare that the research was conducted in the absence of any commercial or financial relationships that could be construed as a potential conflict of interest.

