# Supplementary material for: Integrating DNA Methylation and Gene Expression Data in the Development of the Soybean-Bradyrhizobium N2-Fixing Symbiosis
Source: Front Microbiol. 2016 Apr 22;7:518. doi: 10.3389/fmicb.2016.00518 (PMC4840208; doi:10.3389/fmicb.2016.00518)
Supplement: Supplementary file 1 [file Table1.docx]

Table S1. Differential DNA methylation for each of the 5 identified motifs along the entire genome (first column), within the symbiosis region (second column) and within genome excluding the symbiosis region (third column). The numerator in each cell is the number of modified sites and the denominator is the total number of sites in the genome, with the percent of modified sites in parenthesis.

| **GANTC** | **Full Genome**  methylated / total sites (%) | **Symbiosis Region**  methylated / total sites (%) | **Genome - Symbiosis Region**  methylated / total sites (%) |
| --- | --- | --- | --- |
| free-living (coding) | 23,406 / 23,427 (99.99) | 2,090 / 2,098 (99.62) | 21,316 / 21,329 (99.93) |
| free-living (UTR) | 9,712 / 9,769 (98.42) | 1,267 / 1,270 (99.76) | 8,445 / 8,499 (99.36) |
| free-living (total) | 33,118 / 33,196 (99.77) | 3,357 / 3,368 (99.67) | 29,761 / 29,828 (99.76) |
| endosymbiont (coding) | 22,910 / 23,427 (97.79) | 2,036 / 2,098 (97.04) | 20,874 / 21,329 (97.87) |
| endosymbiont (UTR) | 9,429 / 9,769 (96.52) | 1,229 / 1,270 (96.77) | 8,200 / 8,499 (96.48) |
| endosymbiont (total) | 32,339 / 33,196 (97.41) | 3,265 / 3,368 (96.97) | 29,074 / 29,828 (97.47) |
| **CCTTGAG** | **Full Genome**  methylated / total sites (%) | **Symbiosis Region**  methylated / total sites (%) | **Genome - Symbiosis Region**  methylated / total sites (%) |
| free-living (coding) | 0 / 1,461 (0) | 0 / 104 (0) | 0 / 1,357 (0) |
| free-living (UTR) | 0 / 239 (0) | 0 / 36 (0) | 0 / 203 (0) |
| free-living (total) | 0 / 1,700 (0) | 0 / 140 (0) | 0 / 1,560 (0) |
| endosymbiont (coding) | 1,186 / 1,461 (81.18) | 76 / 104 (73.08) | 1,110 / 1,357 (81.80) |
| endosymbiont (upstream) | 148 / 239 (61.93) | 23 / 36 (63.89) | 125 / 203 (61.58) |
| endosymbiont (total) | 1,334 / 1,700 (78.47) | 99 / 140 (70.71) | 1,235 / 1,560 (79.17) |
| **CRAGGAT** | **Full Genome**  methylated / total sites (%) | **Symbiosis Region**  methylated / total sites (%) | **Genome - Symbiosis Region**  methylated / total sites (%) |
| free-living (coding) | 3,542 / 3,543 (99.97) | 178 / 178 (100.00) | 3,364 / 3,365 (99.97) |
| free-living (upstream) | 461 / 462 (99.78) | 78 / 78 (100.00) | 383 / 384 (99.74) |
| free-living (total) | 4,003 / 4,005 (99.95) | 256 / 256 (100.00) | 3,747 / 3,749 (99.94) |
| endosymbiont (coding) | 3,111 / 3,543 (87.81) | 145 / 178 (81.46) | 2,966 / 3,365 (88.14) |
| endosymbiont (upstream) | 390 / 462 (84.42) | 67 / 78 (85.90) | 323 / 384 (84.11) |
| endosymbiont (total) | 3,501 / 4,005 (87.42) | 212 / 256 (82.81) | 3,289 / 3,749 (87.73) |
| **GAGA(N)_6_RTG** | **Full Genome**  methylated / total sites (%) | **Symbiosis Region**  methylated / total sites (%) | **Genome - Symbiosis Region**  methylated / total sites (%) |
| free-living (coding) | 1,513 / 1,513 (100.00) | 101 / 101 (100.00) | 1,412 / 1,412 (100.00) |
| free-living (upstream) | 567 / 567 (100.00) | 76 / 76 (100.00) | 493 / 493 (100.00) |
| free-living (total) | 2,080 / 2,080 (97.25) | 177 / 177 (100.00) | 1,903 / 1,903 (100.00) |
| endosymbiont (coding) | 1,257 / 1,513 (83.08) | 77 / 101 (76.23) | 1,180 / 1,412 (83.57) |
| endosymbiont (upstream) | 459 / 567 (80.95) | 52 / 76 (68.42) | 404 / 493 (81.95) |
| endosymbiont (total) | 1,716 / 2,080 (82.50) | 129 / 177 (72.88) | 1,584 / 1,903 (82.24) |
| **CAY(N)_6_TCTC** | **Full Genome**  methylated / total sites (%) | **Symbiosis Region**  methylated / total sites (%) | **Genome - Symbiosis Region**  methylated / total sites (%) |
| free-living (coding) | 1,788 / 1,789 (99.94) | 117 / 117 (100) | 1,671 / 1,672 (99.94) |
| free-living (upstream) | 291 / 291 (100) | 58 / 58 (100) | 233 / 233 (100) |
| free-living (total) | 2,080 / 2,080 (100) | 175 / 175 (100) | 1,905 / 1,905 (100) |
| endosymbiont (coding) | 1,558 / 1,789 (87.09) | 86 / 117 (73.50) | 1,472 / 1,672 (88.04) |
| endosymbiont (upstream) | 231 / 291 (79.38) | 42 / 58 (72.41) | 189 / 233 (81.12) |
| endosymbiont (total) | 1,789 / 2,080 (65.83) | 128 / 175 (73.14) | 1,904 / 1,905 (99.95) |
