# Supplementary material for: Integrating DNA Methylation and Gene Expression Data in the Development of the Soybean-Bradyrhizobium N2-Fixing Symbiosis
Source: Front Microbiol. 2016 Apr 22;7:518. doi: 10.3389/fmicb.2016.00518 (PMC4840208; doi:10.3389/fmicb.2016.00518)
Supplement: Supplementary file 2 [file Table2.docx]

**Table S2. Integration of Differential Methylation and Gene Expression**. Column 2 - the number of genes with increased expression in the free-living and and endosymbiont whole genome and symbiosis region; column 3 - genes from column 2 which also have differential methylation; column 4 - genes from column 3 with down methylation in the respective treatment; and column 4 - genes from column 3 with down methylation in their 5’-UTR region. Numbers in parentheses represent proportion of the previous column.

| **GANTC** | **Genes with Increased Expression**  (% of total) | **Differentially Methylated Genes**  (% of differentially expressed) | **Genes with Reduced Methylation**  (% of differentially methylated and expressed) | **Reduced Methylation in 5’-UTR**  (% of genes with reduced methylation) |
| --- | --- | --- | --- | --- |
| **Bacteroid** | 800 / 8,220 (9.73%) | 60 / 800 (7.50%) | 52 / 124 (41.93%) | 9 / 93 (9.68%) |
| **Bacteroid (symbiosis region)** | 200 / 583 (34.31%) | 13/ 200 (6.50%) | 11 / 44 (25.00%) | 2 / 25 (8.00%) |
| **Culture** | 1,382 / 8,220 (16.81%) | 58 / 1,382 (4.20%) | 5 / 128 (3.90%) | 3 / 9 (33.33%) |
| **Culture**  **(symbiosis region)** | 27 / 583 (4.63%) | 3 / 27 (4.63%) | 1 / 3 (33.33%) | 0 / 1 (0%) |
| **CCTTGAG** |  |  |  |  |
| **Bacteroid** | 800 / 8,220 (9.73%) | 95 / 800 (11.88%) | 0 / 97 (0%) | 0 / 0 |
| **Bacteroid (symbiosis region)** | 200 / 583 (34.31%) | 21 / 200 (10.50%) | 0 / 21 (0%) | 0 / 0 |
| **Culture** | 1,382 / 8,220 (16.81%) | 212 / 1,382 (15.34%) | 212 / 212 (100.00%) | 20 / 212 (9.43%) |
| **Culture**  **(symbiosis region)** | 27 / 583 (4.63%) | 2 / 27 (7.41%) | 2 / 2 (100%) | 1 / 2 (50.00%) |
| **CRAGGAT** |  |  |  |  |
| **Bacteroid** | 800 / 8,220 (9.73%) | 62 / 800 (7.75%) | 62 / 62 (100.00%) | 11 / 62 (17.74%) |
| **Bacteroid (symbiosis region)** | 200 / 583 (34.31%) | 17/ 200 (16.00%) | 17/ 17 (100.00%) | 6 / 17 (35.30%) |
| **Culture** | 1,382 / 8,220 (16.81%) | 64 / 1,382 (4.63%) | 0 / 64 (0.00%) | 0 / 0 |
| **Culture**  **(symbiosis region)** | 27 / 583 (4.55%) | 1 / 27 (3.70%) | 0 / 1 (0.00 %) | 0 / 0 |
| **GAGA(N)_6_RTG** |  |  |  |  |
| **Bacteroid** | 800 / 8,220 (9.73%) | 40 / 800 (0.50%) | 40 / 40 (100.00%) | 12 / 40 (30.0%) |
| **Bacteroid (symbiosis region)** | 200 / 583 (34.31%) | 16 / 200 (8.00%) | 16 / 16 (100.00%) | 7 / 16 (43.75%) |
| **Culture** | 1,382 / 8,220 (16.81%) | 53 / 1,382 (7.42%) | 0 / 101 (0.30%) | 0 / 3 (33.33%) |
| **Culture**  **(symbiosis region)** | 27 / 583 (4.63%) | 0 / 27 (0%) | 0 / 2 (0%) | 0 / 0 |
| **CAY(N)_6_TCTC** |  |  |  |  |
| **Bacteroid** | 800 / 8,220 (9.73%) | 36 / 800 (4.50%) | 36 / 36 (100%) | 4 / 36 (11.11%) |
| **Bacteroid (symbiosis region)** | 200 / 583 (34.31%) | 19 / 200 (9.50%) | 19 / 19 (100%) | 3 / 19 (15.79%) |
| **Culture** | 1,382 / 8,220 (16.81%) | 41 / 1,382 (4.99%) | 0 / 41 (0%) | 0 / 0 |
| **Culture**  **(symbiosis region)** | 27 / 583 (4.63%) | 1 / 27 (3.70%) | 0 / 1 (0%) | 0 / 0 |
